# Interpreting the triglyceride–glucose index and its derived indices in metabolic dysfunction-associated steatotic liver disease: clinical utility and evidence gaps

**DOI:** 10.3389/fmed.2026.1857612

**Published:** 2026-08-11

**Authors:** Longzhou Chen, Yucai Tang, Hao Guo, Wencai Jiang, Xuejun Deng

**Affiliations:** Department of Cardiology, Suining Central Hospital, Suining, Sichuan, China

**Keywords:** cardiometabolic risk, insulin resistance, metabolic dysfunction-associated steatotic liver disease, non-invasive risk assessment, triglyceride–glucose index

## Abstract

Metabolic dysfunction-associated steatotic liver disease (MASLD) is common, yet early disease often remains unrecognized. The triglyceride–glucose (TyG) index, calculated from fasting triglyceride and glucose concentrations, has emerged as an accessible surrogate marker of metabolic dysfunction. This review examines the clinical interpretation, biological context, and current limitations of the TyG index and its anthropometric and inflammatory derivatives in MASLD. Current evidence links higher TyG-related measures to prevalent steatosis, fibrosis markers, and selected extrahepatic outcomes. Indices incorporating body mass index, waist circumference, or waist-to-height ratio may improve risk discrimination in some settings, particularly when body-fat distribution is informative. However, their performance varies according to population characteristics, disease definition, reference standard, and clinical purpose. TyG is not a direct measure of hepatic or peripheral insulin resistance, liver inflammation, or fibrosis. It should therefore be considered a metabolic risk marker rather than a stand-alone diagnostic test for MASLD or MASH. Important limitations include the predominance of cross-sectional evidence, heterogeneity among NAFLD, MAFLD, and MASLD definitions, variable cutoff values, and component overlap between TyG-derived indices and cardiometabolic diagnostic criteria. Evidence for liver-specific hard outcomes remains limited. Future studies should directly compare TyG-related markers with established metabolic and liver-directed tests in diverse prospective cohorts, using standardized repeated measurements and outcomes including progressive fibrosis, cirrhosis, hepatocellular carcinoma, and liver-related death. At present, TyG-related measures may complement, but cannot replace, liver-specific assessment.

## 1 Introduction

Metabolic dysfunction-associated steatotic liver disease (MASLD) is characterized by hepatic steatosis in the setting of cardiometabolic dysfunction and affects approximately one-third of adults worldwide (1). It is an increasingly important contributor to advanced chronic liver disease, including cirrhosis and hepatocellular carcinoma (2). The clinical spectrum extends from steatosis to metabolic dysfunction-associated steatohepatitis (MASH), progressive fibrosis, cirrhosis, and hepatocellular carcinoma; fibrosis stage is the major determinant of liver-related outcomes (3). Early identification of individuals at risk of progressive disease is therefore clinically important. Table 1 summarizes the current diagnostic criteria for MASLD based on the 2023 international consensus.

**Table 1:** Diagnostic criteria for metabolic dysfunction-associated steatotic liver disease (MASLD) (2023 consensus).

Component	Adult criterion	Interpretation
Evidence of hepatic steatosis	Hepatic steatosis identified by imaging or histology	In advanced fibrosis or cirrhosis, steatosis may be absent; classification then requires clinical judgment based on cardiometabolic risk factors and exclusion of alternative explanations.
Diagnostic requirement	Hepatic steatosis plus at least one cardiometabolic risk factor	The presence of one qualifying cardiometabolic risk factor is sufficient for MASLD classification.
1. Adiposity	Body mass index ≥25 kg/m^2^, or ≥23 kg/m^2^ in Asian populations; alternatively, waist circumference >94 cm in men or>80 cm in women, or an ethnicity-adjusted equivalent	BMI and waist circumference should not be treated as interchangeable measures of adiposity. Waist-based measures may be more informative when central adiposity is clinically relevant.
2. Dysglycaemia or type 2 diabetes	Fasting plasma glucose ≥5.6 mmol/L (100 mg/dL), 2-h post-load glucose ≥7.8 mmol/L (140 mg/dL), HbA1c ≥ 5.7%, established type 2 diabetes, or treatment for type 2 diabetes	This criterion captures prediabetes, overt diabetes, and treated diabetes.
3. Elevated blood pressure	Blood pressure ≥130/85 mmHg or use of antihypertensive medication	Either measured elevation or pharmacological treatment fulfils this criterion.
4. Hypertriglyceridaemia	Plasma triglycerides ≥1.70 mmol/L (150 mg/dL) or lipid-lowering treatment	Triglyceride elevation is directly relevant to the biological interpretation of the TyG index.
5. Low HDL-cholesterol	HDL-cholesterol ≤1.0 mmol/L (40 mg/dL) in men or ≤1.3 mmol/L (50 mg/dL) in women, or lipid-lowering treatment	Sex-specific thresholds should be retained.
Assessment of additional causes	Alcohol exposure and other causes of steatotic liver disease should be assessed	Coexisting drivers do not necessarily exclude metabolic dysfunction. They may indicate a combination aetiology, such as MetALD, rather than pure MASLD.

MASLD is diagnosed in adults with hepatic steatosis and at least one cardiometabolic risk factor. NAFLD and MAFLD should be retained when reporting the original terminology and diagnostic definitions of other cardiometabolic risk factors earlier studies. The table summarizes adult criteria; paediatric definitions require age-specific thresholds. Adapted from the multisociety Delphi consensus statement (4).

Throughout this review, terminology follows the 2023 multisociety consensus (4). MASLD denotes hepatic steatosis with at least one cardiometabolic risk factor, whereas MASH is reserved for the inflammatory disease state. Earlier studies are cited under their original NAFLD or MAFLD designations. Differences in metabolic entry criteria, alcohol thresholds, and reference standards mean that these cohorts are not fully interchangeable with contemporary MASLD populations. This distinction is particularly important when interpreting TyG-related evidence, because the apparent performance of a marker depends on the population and disease definition used. Although MASLD is common and clinically consequential, early disease is frequently silent, and available screening approaches have important limitations. Liver enzyme tests lack sensitivity, and ultrasonography may fail to detect mild steatosis (3). As a result, many individuals are not identified until disease has progressed beyond its earliest stage. Current frameworks based on overt metabolic abnormalities may also miss people with subclinical dysfunction, including some individuals with lean MASLD or borderline metabolic profiles, who may still be at risk of progression (5).

Insulin resistance (IR) is a central, although not exclusive, mechanism in MASLD (6, 7). The hyperinsulinemic-euglycemic clamp is the reference method for assessing insulin sensitivity, but its complexity, duration, and cost limit routine clinical use (7). The triglyceride-glucose (TyG) index, calculated from fasting triglyceride and glucose concentrations, was developed as an accessible surrogate of systemic insulin resistance and has been compared directly with clamp-based measurements (8, 9). Observational studies and meta-analyses have linked higher TyG values with prevalent and incident steatotic liver disease, although the strength of association varies across populations and diagnostic definitions (10–13). Anthropometric, inflammatory, and longitudinal derivatives have subsequently been proposed to incorporate body-fat distribution, inflammation, or cumulative metabolic exposure. Their potential value lies in complementary risk enrichment rather than direct diagnosis or staging of MASLD.

**Figure 1:**
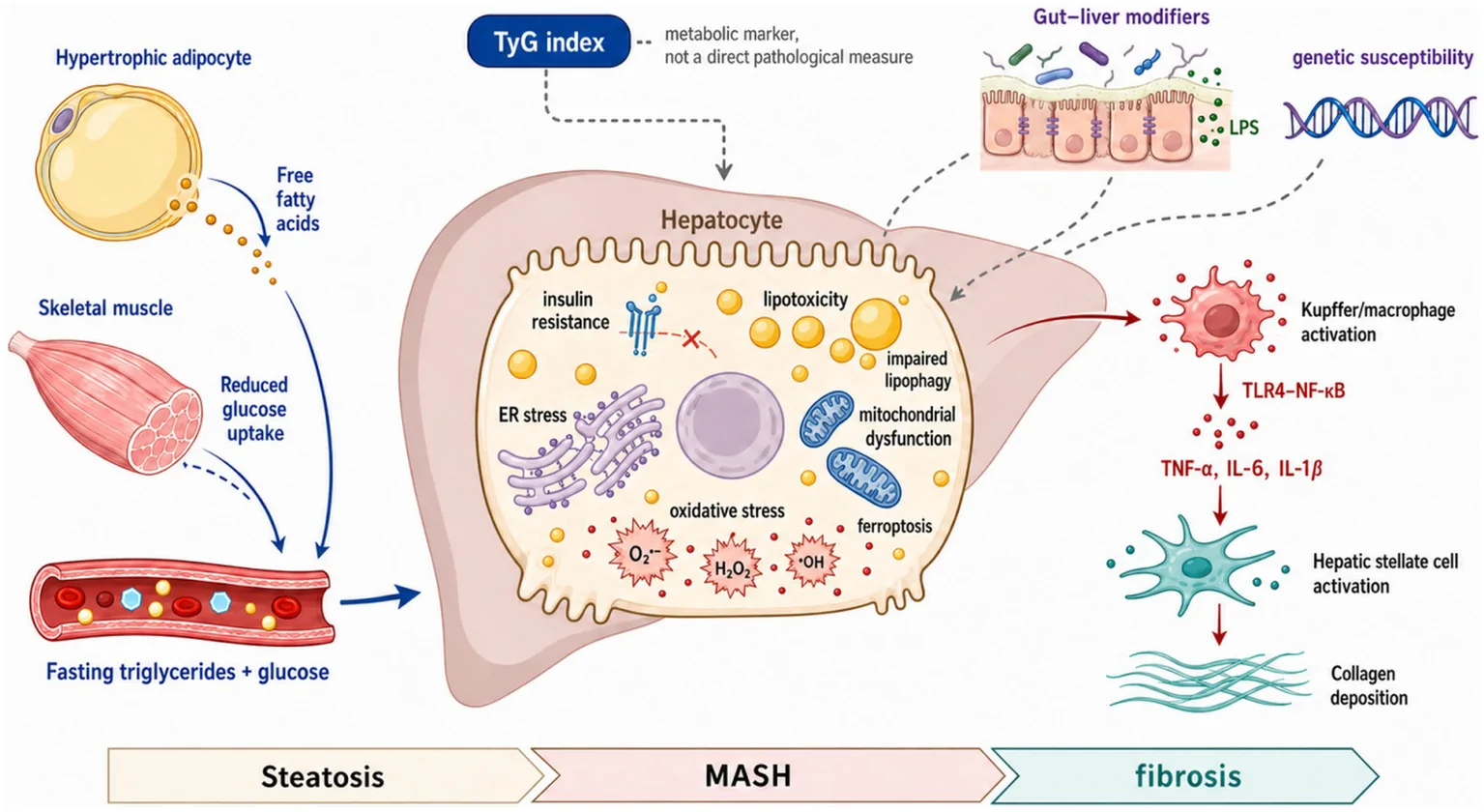
Biological context for the association between the triglyceride–glucose (TyG) index and MASLD progression. Insulin resistance across adipose tissue, skeletal muscle, and the liver increases free-fatty-acid flux and perturbs triglyceride and glucose metabolism; fasting triglyceride and glucose concentrations are combined in the TyG index. In hepatocytes, altered lipid flux promotes lipotoxicity, impaired lipophagy, endoplasmic reticulum stress, mitochondrial dysfunction, oxidative stress, and ferroptosis. Gut-derived lipopolysaccharide and genetic susceptibility can modify these processes. Hepatocellular stress promotes Kupffer cell/macrophage activation and TLR4–NF-κB-mediated cytokine signalling, leading to hepatic stellate-cell activation and collagen deposition as disease progresses from steatosis to MASH and fibrosis. TyG is depicted as a circulating metabolic risk marker rather than a direct measure of tissue-specific insulin resistance, hepatic inflammation, or fibrosis. Solid arrows indicate proposed biological directions, whereas dashed arrows denote indirect associations or modifying influences rather than direct causality. Blue, red, and teal arrows represent systemic metabolic, inflammatory, and fibrogenic processes, respectively.

However, these markers have important limitations. Much of the available evidence is cross-sectional, reported cutoff values are heterogeneous, and external validation across sex, age, ethnicity, diabetes status, and lean phenotypes remains limited (14). Their ability to predict liver-specific hard outcomes, including progressive fibrosis, cirrhosis, hepatocellular carcinoma, and liver-related death, is also uncertain. This review therefore critically evaluates the biological interpretation, clinical utility, and limitations of TyG-related indices in MASLD and considers the evidence required before their use in routine risk-stratification pathways. Figure 1 summarizes the biological context linking the systemic metabolic dysfunction captured by TyG to hepatocellular stress, inflammation, and fibrogenic progression.

## 2 TyG and its derivatives

### 2.1 The original TyG index

The TyG index, defined as ln [fasting triglycerides (mg/dL) × fasting plasma glucose (mg/dL)/2], was introduced as a practical surrogate marker of insulin resistance (IR) (8). Its appeal lies in its reliance on routine fasting lipid and glucose measurements, without the need for insulin assays or complex metabolic testing. Studies comparing TyG with the hyperinsulinemic-euglycemic clamp support its use as an accessible indicator of metabolic dysfunction in selected populations (9). However, TyG does not directly quantify either hepatic or peripheral IR, and it does not provide direct evidence of hepatic steatosis, MASH, or fibrosis. Its biological interpretation should therefore remain distinct from the diagnosis and staging of liver disease. Studies conducted under earlier NAFLD and MAFLD definitions have consistently linked higher TyG values with prevalent steatosis and with incident fatty liver disease (10–13). These associations support its use for metabolic risk identification, but their magnitude depends on the underlying population, disease definition, and reference standard. Reported thresholds should consequently be interpreted as study-specific decision values rather than universal diagnostic cutoffs. In a direct comparison of insulin-resistance markers among dysmetabolic adults, TyG showed lower discriminatory performance for MASLD than HOMA-IR and lipid accumulation product, although it remained easier to calculate in routine practice (15). Accordingly, an elevated TyG value may help identify individuals who warrant more careful metabolic and liver-focused assessment. It should not be used alone to diagnose MASLD or to replace imaging-based assessment of hepatic steatosis. A normal TyG value should likewise not be interpreted as excluding MASLD, particularly in people with lean phenotypes, treated dyslipidaemia, or other metabolic risk factors not fully captured by fasting triglyceride and glucose levels.

### 2.2 Anthropometric derivatives

Combining the TyG index with anthropometric measures addresses a specific limitation of the original index: fasting triglycerides and glucose do not describe body-fat distribution. TyG-BMI incorporates overall adiposity, whereas TyG-WC and TyG-WHtR place greater emphasis on central adiposity. These derivatives should not be regarded as interchangeable because each captures a different component of metabolic risk. Two recent meta-analyses reported an AUC of 0.83 for TyG-BMI but reached this estimate from partly different evidence bases. Boushehri et al. included 35 studies and 339,087 participants and reported pooled sensitivity and specificity of 0.79 and 0.76, respectively; Du et al. (16) included 29 studies and reported corresponding estimates of 0.81 and 0.72 (17). Both analyses showed substantial heterogeneity in disease nomenclature, populations, thresholds, and reference standards. In United States adolescents, TyG-WC had the highest discriminatory performance for MAFLD, while TyG-BMI and TyG-WHtR also performed well (18). These findings are consistent with the view that central adiposity adds clinically relevant information beyond fasting glucose and triglycerides. However, the apparent advantage of a derivative may partly reflect the prevalence of obesity, diabetes, and metabolic dysfunction in the study population. This issue is particularly relevant in lean MASLD and older adults. In individuals with normal BMI, adiposity may be underestimated when fat distribution is not considered, and waist-based derivatives may identify metabolically unhealthy individuals who would not be recognized by BMI alone (19). Nevertheless, current evidence does not establish TyG-WC or TyG-WHtR as the preferred marker for lean adult MASLD. BMI may also be less informative in older adults because sarcopenia and changes in body composition can obscure adiposity. Sex-specific findings require similar caution. Both meta-analyses suggested better TyG-BMI discrimination in women, but these subgroup observations should not be attributed solely to sex-related fat distribution without direct mechanistic evidence (16, 17). No TyG-derived index can currently be recommended as universally superior.

### 2.3 Emerging derivatives

A single TyG measurement captures metabolic status at one time point, whereas MASLD develops within a changing metabolic environment. Longitudinal approaches, including cumulative TyG exposure and trajectory analysis, may therefore provide information unavailable from a baseline value alone. Persistently higher TyG trajectories have been associated with incident lean NAFLD and new-onset MASLD (20, 21). These findings support repeated assessment but do not establish a uniform trajectory threshold or validated monitoring schedule. Repeated fasting measurements should use consistent laboratory methods and prespecified intervals, with changes in body weight, medication use, alcohol exposure, diabetes status, and intercurrent illness considered explicitly. Composite measures have also been developed to incorporate additional biological domains. The hs-CRP–TyG index has shown cross-sectional associations with NAFLD and liver fibrosis (22). In two nationwide prospective cohorts, CRP–TyG was also associated with all-cause and cardiovascular mortality among participants with MASLD (23). These observations extend prognostic associations beyond cross-sectional liver phenotypes, but they do not establish prediction of liver-specific hard outcomes. Interpretation is further complicated because hs-CRP and TyG may capture overlapping consequences of metabolic inflammation. Emerging derivatives should therefore remain exploratory until incremental discrimination, calibration, clinical utility, and external validation have been demonstrated.

### 2.4 Clinical interpretation and selection of TyG-related indices

The clinical value of the TyG index does not lie in a universal cutoff or in its use as a stand-alone diagnostic test. Rather, it lies in its ability to identify an adverse metabolic milieu using measurements that are routinely available in clinical practice. A single TyG value may help to enrich the population at risk, but it neither demonstrates hepatic steatosis nor replaces liver-directed assessment by imaging or validated non-invasive tests. Its role should therefore be considered complementary to, rather than interchangeable with, established markers of insulin resistance and liver disease.

The choice of a TyG-related index should be guided by the clinical question and by the characteristics of the population being assessed. TyG-BMI incorporates overall adiposity, whereas TyG-WC and TyG-WHtR place greater emphasis on central fat distribution. Two recent meta-analyses found moderate-to-good TyG-BMI discrimination but substantial heterogeneity in disease definitions, reference standards, thresholds, and participant characteristics (16, 17). In United States. adolescents, TyG-WC showed the highest discriminatory performance for MAFLD identified using controlled attenuation parameter, while TyG-BMI and TyG-WHtR also performed well (18). These findings support the potential value of adiposity-integrated indices in selected populations, particularly when central obesity is clinically relevant. They do not, however, justify a universal preference for one derivative or direct extrapolation to lean adult MASLD. Comparative data also argue against presenting TyG as a replacement for other insulin-resistance markers. In dysmetabolic adults, TyG discriminated MASLD less well than HOMA-IR and lipid accumulation product, although its lower cost and lack of an insulin measurement remain practical advantages (15). Inflammatory composites require similar caution. The hs-CRP–TyG index showed better cross-sectional discrimination of NAFLD and significant fibrosis than TyG or hs-CRP alone in NHANES participants (22). However, because this index combines metabolic and inflammatory signals, its apparent advantage may partly reflect overlapping biological information rather than a distinct pathogenic pathway. Its clinical relevance requires prospective validation. Longitudinal evidence is emerging but remains limited. In a health-management cohort, a high-stable TyG trajectory was associated with incident lean NAFLD, indicating that repeated metabolic assessment may be more informative than a single measurement in some individuals (20). Likewise, data from the United Kingdom Biobank linked higher TyG-related indices, particularly TyG-WC and TyG-WHtR, with subsequent cardiovascular disease and mortality among participants with MASLD (24). These outcomes are clinically important, but they should not be conflated with liver-specific endpoints. Evidence remains insufficient to establish whether TyG-related indices predict progression to advanced fibrosis, cirrhosis, hepatocellular carcinoma, or liver-related death.

Accordingly, TyG-related indices are best viewed as context-dependent metabolic risk markers. Their use should be tailored to the target population, the available reference tests, and the intended outcome. Population-specific thresholds, direct head-to-head comparisons, and prospective studies with liver-related hard endpoints are still required before these indices can be incorporated into routine MASLD risk-stratification pathways (Table 2).

**Table 2:** Clinically interpretable evidence for the TyG index and related indices in MASLD and related steatotic liver disease.

Clinical question	Index	Study design and population	Disease definition/reference standard	Main quantitative finding	Appropriate interpretation and main limitation
Comparative identification of MASLD	TyG, HOMA-IR, LAP, VAI, TG/HDL-C	Prospective Plinio Study; dysmetabolic adults; *n* = 772	MASLD assessed by ultrasound plus cardiometabolic criteria	For MASLD identification, AUC was 0.723 for TyG, versus 0.792 for HOMA-IR and 0.787 for LAP.	TyG was associated with MASLD but was not the best-performing marker in this cohort. It should be presented as an accessible surrogate marker rather than a replacement for HOMA-IR or imaging. (15)
Pooled diagnostic performance of an adiposity-derived index	TyG-BMI	Systematic review and meta-analysis; 29 studies	Studies used heterogeneous NAFLD/MAFLD/MASLD definitions and reference standards	Pooled sensitivity: 0.81; specificity: 0.72; AUC: 0.83.	TyG-BMI shows moderate-to-good discriminatory performance, but heterogeneity in disease nomenclature, populations, and diagnostic methods precludes a universal cutoff. (16)
Pediatric and non-obese subgroup screening	TyG, TyG-BMI, TyG-WC, TyG-WHtR	Cross-sectional NHANES 2017–2020 study; United States. adolescents; *n* = 532	MAFLD defined using CAP-detected steatosis plus metabolic criteria	TyG-WC had the highest AUC: 0.923 (95% CI 0.900–0.947); TyG-BMI: 0.917; TyG-WHtR: 0.915; original TyG: 0.673.	Adiposity-integrated indices outperformed TyG in this adolescent dataset, including non-obese subgroups. This must not be generalized directly to adult lean MASLD without validation. (18)
Inflammation-integrated index and fibrosis screening	CTI (hs-CRP–TyG index)	Cross-sectional NHANES 2017–2020 study; United States adults; *n* = 3,488	NAFLD: CAP ≥274 dB/m; significant fibrosis: liver stiffness ≥8.2 kPa	CTI AUC was 0.756 for NAFLD and 0.702 for significant fibrosis; both exceeded TyG and hs-CRP alone.	This is cross-sectional evidence for an inflammation-metabolic composite, not prospective proof of fibrosis progression. CTI may also overlap biologically with inflammatory pathways already represented by CRP. (22)
Longitudinal risk in lean individuals	TyG trajectory	Health management cohort; lean adults; *n* = 1,109	Incident lean NAFLD assessed by abdominal ultrasound	Compared with the low-stable trajectory, the high-stable TyG trajectory was associated with incident lean NAFLD: HR 2.668 (95% CI 1.098–6.484).	Supports longitudinal TyG assessment in lean populations, but the study used the older NAFLD definition, ultrasound, a short follow-up period, and a single-center cohort. (20)
Extrahepatic prognosis in established MASLD	TyG, TyG-BMI, TyG-WC, TyG-WHtR	UK Biobank prospective cohort; MASLD participants; *n* = 97,331; median follow-up 13.56 years	MASLD defined at baseline in United Kingdom Biobank	For incident CVD, fourth-versus-first-quartile HRs were 1.19 for TyG, 1.35 for TyG-BMI, 1.33 for TyG-WC, and 1.39 for TyG-WHtR. TyG-WC and TyG-WHtR improved CVD/mortality prediction.	Supports association with cardiovascular and all-cause mortality outcomes, not liver-specific hard outcomes such as cirrhosis, HCC, or liver-related death. (24)

AUC, area under the receiver operating characteristic curve; CAP, controlled attenuation parameter; CTI, C-reactive protein–triglyceride–glucose index; CVD, cardiovascular disease; HOMA-IR, homeostasis model assessment of insulin resistance; LAP, lipid accumulation product; TyG, triglyceride–glucose index; VAI, visceral adiposity index; WC, waist circumference; WHtR, waist-to-height ratio. NAFLD and MAFLD are retained when reporting the original terminology used by individual studies and should not be regarded as fully interchangeable with MASLD. Reported cutoff values are study-specific and are not recommended for universal clinical use.

**Figure 2:**
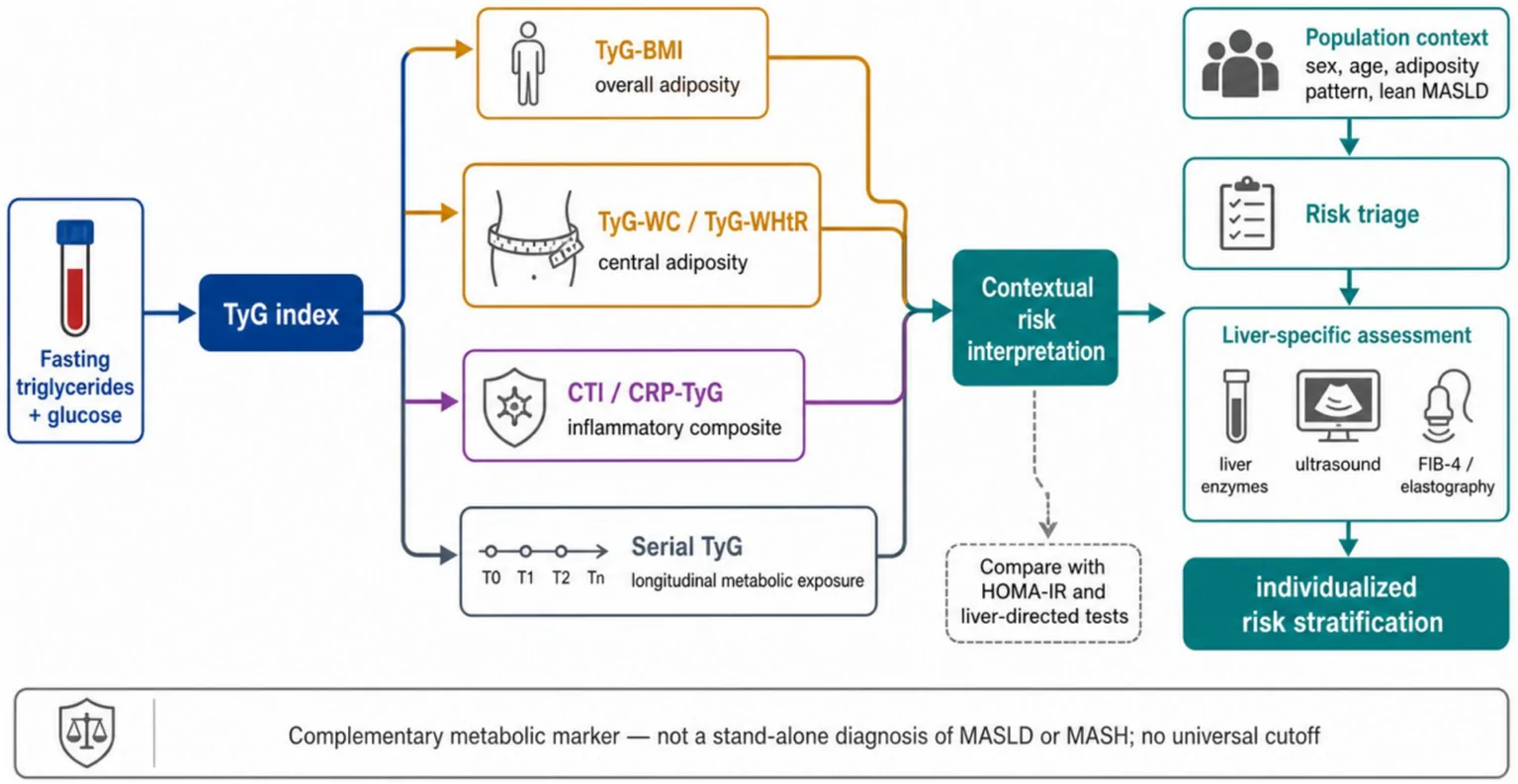
Context-specific selection and interpretation of TyG-related indices in MASLD. The original TyG index reflects fasting triglyceride–glucose metabolism, whereas TyG-BMI, TyG-WC, and TyG-WHtR incorporate overall or central adiposity and CTI/hs-CRP–TyG adds an inflammatory component. Selection should be guided by population characteristics, clinical purpose, and available liver-directed tests. TyG-related indices should be interpreted as complementary metabolic risk markers rather than stand-alone tests for MASLD, MASH, or fibrosis.

Figure 2 provides a context-specific framework for selecting and interpreting TyG-related indices. These measures may enrich metabolic risk assessment when considered alongside population characteristics, body-fat distribution, and liver-directed evaluation; however, they should not be used as stand-alone diagnostic or staging tests for MASLD or MASH.

## 3 Pathophysiological interpretation of TyG-related risk in MASLD

An elevated TyG index should be interpreted as a composite metabolic signal rather than as a molecular mediator of liver injury. Because the index combines fasting triglycerides and glucose, it may reflect the metabolic conditions that accompany insulin resistance, dysregulated lipid handling, and hepatic fat accumulation. However, an association between TyG and MASLD does not establish that TyG itself directly causes steatosis, inflammation, or fibrosis. Its value lies in the metabolic information it captures, whereas disease diagnosis and staging still require liver-directed assessment. The biological interpretation of TyG-related risk is therefore necessarily indirect. Insulin resistance can increase hepatic substrate availability and *de novo* lipogenesis, while lipotoxic intermediates may promote mitochondrial dysfunction, endoplasmic reticulum stress, oxidative stress, and inflammatory signalling. Genetic susceptibility, adipose-tissue dysfunction, and gut–liver axis perturbation further modify this pathway and may partly explain why similar TyG values do not confer identical risk across individuals. These processes can converge on hepatocyte injury and hepatic stellate-cell activation, thereby linking steatosis to MASH and fibrosis (Figure 1).

The following sections distinguish the mechanisms that TyG may plausibly reflect from those that it cannot directly measure. This distinction is important when interpreting molecular studies, Mendelian-randomization analyses, and multi-omics findings. Such evidence may strengthen biological plausibility, but it does not by itself convert TyG into a causal marker or a direct measure of any single pathogenic pathway.

### 3.1 Insulin resistance as a metabolic Nexus

Insulin resistance is a central metabolic nexus in MASLD, but it should not be regarded as the sole initiating event.

Its importance lies in the way it links systemic nutrient excess to altered glucose handling, adipose-tissue lipolysis, hepatic lipid accumulation, and inflammatory susceptibility (15, 25). Genetic predisposition, adipokine imbalance, dietary exposures, and gut–liver axis dysfunction may modify these processes before or alongside overt insulin resistance. This broader framework is particularly relevant to lean MASLD, in which clinically apparent obesity may not adequately reflect underlying metabolic risk. Peripheral and hepatic insulin resistance make distinct contributions to this process. In skeletal muscle, impaired insulin signalling reduces glucose disposal and contributes to higher circulating glucose concentrations. In adipose tissue, inadequate suppression of lipolysis increases the release of free fatty acids into the circulation. The liver takes up a proportion of these fatty acids and uses them for triglyceride synthesis and very-low-density-lipoprotein production. Dysregulation of hormone-sensitive lipase and other pathways involved in lipid turnover can further amplify this flux (26, 27). Hepatic insulin resistance has additional consequences. Impaired suppression of gluconeogenesis contributes to fasting hyperglycaemia, while persistent lipogenic signalling can promote hepatic triglyceride accumulation despite systemic insulin resistance (28). The coexistence of higher fasting glucose and triglycerides provides the biological rationale for the TyG index. Nevertheless, TyG cannot distinguish hepatic from peripheral insulin resistance, nor can it identify the relative contribution of adipose tissue, skeletal muscle, or liver to an individual’s metabolic phenotype. It should therefore be interpreted as an integrated surrogate of metabolic disturbance rather than as a tissue-specific measure of insulin resistance. This distinction has practical implications for MASLD risk assessment. TyG may be informative when interpreted alongside body-fat distribution, glycaemic status, and liver-directed tests. It cannot, however, determine whether hepatic steatosis is driven predominantly by insulin resistance, genetic susceptibility, altered adipokine signalling, or gut-derived inflammatory stimuli.

### 3.2 Lipotoxicity, organelle stress, and hepatocellular injury

Hepatic triglyceride accumulation is not, by itself, synonymous with hepatocellular injury. Triglycerides may initially provide a relatively inert storage form for excess fatty acids. Injury becomes more likely when lipid influx, *de novo* lipogenesis, oxidation, and export are no longer balanced. Under these conditions, hepatocytes accumulate bioactive lipid species, including diacylglycerols, ceramides, and free cholesterol. These intermediates can disrupt insulin signalling, membrane integrity, and organelle function, thereby converting metabolic overload into lipotoxic stress (29, 30).

The endoplasmic reticulum and mitochondria are central to this transition. Lipid excess can perturb endoplasmic-reticulum homeostasis and activate the unfolded-protein response. When this response is prolonged or unresolved, it may promote inflammatory signalling, redox imbalance, and hepatocyte apoptosis. In parallel, excessive fatty-acid delivery increases mitochondrial workload. Incomplete *β*-oxidation and electron leakage can enhance reactive oxygen species production, while persistent mitochondrial dysfunction limits the cell’s capacity to adapt to metabolic stress (31). These processes are interdependent: endoplasmic-reticulum stress can impair mitochondrial function, and mitochondrial reactive oxygen species can further aggravate endoplasmic-reticulum stress. Autophagic pathways provide an additional layer of metabolic control. Lipophagy facilitates the clearance of lipid droplets and can supply fatty acids for regulated mitochondrial oxidation. Impaired lipophagy may therefore promote intracellular lipid retention, oxidative stress, and hepatocellular injury (32). Lipid peroxidation represents a further route from metabolic stress to cell damage. In experimental MASH models, iron-dependent lipid peroxidation, or ferroptosis, has been implicated as a potential mechanism of hepatocyte injury. However, most current evidence remains preclinical, and its relevance to risk stratification in patients with MASLD is not yet established (33–35).

These events provide biological plausibility for the association between higher TyG values and more severe liver disease. They do not imply that TyG directly measures ceramides, endoplasmic-reticulum stress, mitochondrial dysfunction, impaired lipophagy, or ferroptosis. The index should instead be viewed as a circulating metabolic signal that may accompany several interconnected pathways of lipotoxic injury.

### 3.3 Inflammation and immune activation

Lipotoxic stress can shift the liver from relatively uncomplicated steatosis toward an inflammatory state. Injured hepatocytes release damage-associated molecular patterns (DAMPs), which activate Kupffer cells and recruit additional innate and adaptive immune cells. Activated hepatic macrophages produce mediators such as tumour necrosis factor-*α*, interleukin-6, and interleukin-1β. These signals promote hepatocyte injury, disturb insulin signalling, and sustain local inflammation (36, 37). The TLR4–NF-κB axis provides an important link between metabolic stress and inflammatory amplification. Excess free fatty acids and gut-derived lipopolysaccharide can activate TLR4 signalling in hepatocytes and resident immune cells. Downstream NF-κB activation promotes the transcription of pro-inflammatory mediators and can reinforce oxidative stress and lipotoxic injury (38, 39). The result is not a linear sequence, but a self-reinforcing network in which lipid overload, inflammation, and cellular stress intensify one another during progression from steatosis to MASH (Figure 1).

TyG is not a direct marker of immune-cell activation, circulating endotoxin, intestinal permeability, or TLR4 signalling. Higher TyG values may coexist with these processes because they share metabolic drivers, but they cannot establish the presence or magnitude of hepatic inflammation in an individual patient. This limitation is particularly relevant when interpreting inflammatory TyG derivatives, such as the hs-CRP–TyG index. Any apparent improvement in discrimination should be evaluated as an incremental predictive effect, rather than as evidence that the composite index directly captures the underlying inflammatory pathway.

### 3.4 Oxidative stress as an amplifying circuit

Oxidative stress arises when reactive oxygen species production exceeds the capacity of cellular antioxidant systems. In MASLD, mitochondrial fatty-acid oxidation is an important source of reactive oxygen species under conditions of sustained nutrient overload. Electron leakage from the respiratory chain, impaired mitochondrial quality control, and disrupted endoplasmic-reticulum–mitochondrial communication can all increase oxidative burden (31, 32). Oxidative stress is therefore not simply a late consequence of steatosis. It can amplify the injury produced by lipotoxicity and organelle dysfunction.

Excessive reactive oxygen species promote lipid peroxidation and generate reactive aldehydes, including malondialdehyde and 4-hydroxynonenal. These products can damage mitochondrial membranes, proteins, and DNA, thereby further compromising cellular energy metabolism and hepatocyte viability (33). Oxidative stress can also reinforce NF-κB-dependent inflammatory signalling, while inflammatory mediators may worsen mitochondrial dysfunction. This bidirectional interaction links metabolic overload to persistent inflammation and provides a route by which steatosis may progress toward MASH and fibrosis ((40, 41); Figure 1).

Oxidative stress also intersects with the ferroptotic pathways discussed above, because lipid peroxidation is a defining feature of ferroptotic cell death. However, the relative contribution of oxidative stress, apoptosis, necroinflammation, and ferroptosis is likely to vary across disease stage and patient phenotype. These mechanisms should not be inferred from TyG values alone. TyG may reflect a metabolic context in which oxidative injury is more likely to occur, but it is not a direct measure of reactive oxygen species, antioxidant capacity, or lipid peroxidation.

### 3.5 Fibrosis as a consequence of persistent injury

Liver fibrosis represents a wound-healing response to persistent hepatocellular injury rather than an inevitable consequence of steatosis. Recurrent lipotoxicity, oxidative stress, inflammatory signalling, and cell death activate hepatic stellate cells (HSCs), the principal extracellular-matrix-producing cells in chronic liver disease (42, 43). Activated HSCs acquire a myofibroblast-like phenotype and produce excessive collagen and other matrix components. Transforming growth factor-*β* and platelet-derived growth factor are major signals in this process, although interactions with macrophages, immune cells, and the surrounding matrix also influence the intensity and reversibility of fibrosis (44, 45).

Fibrosis stage remains the strongest predictor of liver-related outcomes in MASLD. This clinical relevance explains the interest in TyG-related markers as potential tools for identifying individuals at increased risk of advanced disease. However, an association between TyG and fibrosis should not be interpreted as evidence of a direct profibrotic effect. Higher TyG values may reflect upstream metabolic conditions that increase susceptibility to lipotoxic injury, inflammation, and HSC activation. Whether TyG or its derivatives can predict fibrosis progression beyond established liver-directed tests remains uncertain and requires prospective validation with clearly defined clinical endpoints.

This distinction is important for clinical interpretation. TyG-related indices may help identify metabolic risk in people who require further liver assessment, but they cannot replace fibrosis staging by validated non-invasive tests, imaging-based elastography, or histology when clinically indicated (Figure 1).

### 3.6 Systemic and genetic modifiers of TyG-related risk

MASLD develops within a systemic metabolic environment rather than through liver-specific mechanisms alone. Adipose-tissue dysfunction can increase the delivery of free fatty acids to the liver and alter adipokine secretion. Reduced adiponectin may weaken insulin-sensitising and anti-inflammatory signalling, whereas increased leptin and other adipose-derived mediators may favour inflammation and fibrogenic responses (46). These changes may help explain why TyG-derived indices that incorporate waist-based measures can provide additional information in some populations. They do not, however, establish that a high TyG value identifies a specific adipokine abnormality in an individual patient.

The gut–liver axis provides a further systemic route through which metabolic stress may influence liver injury. Gut dysbiosis and impaired intestinal barrier function can increase the delivery of microbial products, including lipopolysaccharide, to the portal circulation. These products may activate hepatic immune cells and TLR4-dependent inflammatory signalling, thereby reinforcing the lipotoxic and inflammatory pathways described above (47). Recent phenotype-stratified analysis has also identified distinct gut microbial and circulating transcriptomic profiles across metabolic phenotypes in MASLD, supporting integrated rather than single-marker interpretation of the gut–liver axis (48). TyG does not directly measure intestinal permeability, microbial composition, endotoxaemia, or TLR4 activation. Any relationship between TyG and gut-derived inflammation should therefore be described as indirect and hypothesis-generating.

Genetic susceptibility may further modify the relationship between metabolic dysfunction and liver injury. Variants affecting hepatic lipid handling, including loci involving PNPLA3, TM6SF2, and GCKR, may influence steatosis severity or progression independently of conventional anthropometric measures. Recent multi-omics analyses have nominated shared genetic and transcriptional signals for TyG and MASLD, but these findings should be interpreted cautiously. Mendelian-randomization and multi-omics results can support biological plausibility, yet they do not prove that elevated TyG is itself a direct causal driver of MASLD (49). Importantly, evidence on interactions between TyG-related indices and common MASLD susceptibility variants remains limited. This is an important gap for future precision-risk studies.

## 4 Limitations of current evidence

The current evidence base is dominated by cross-sectional analyses. Such studies can identify associations between TyG-related indices and steatosis, fibrosis markers, or prevalent MASLD, but they cannot establish temporal sequence or causality. Although genetic epidemiology and Mendelian-randomization analyses have generated hypotheses regarding potential links between TyG and MASLD, these results remain vulnerable to assumptions concerning instrument validity, pleiotropy, and phenotype definition. They should therefore be interpreted as supportive of biological plausibility rather than as definitive proof that TyG elevation directly causes MASLD (49, 50).

Heterogeneity in disease nomenclature and diagnostic reference standards further limits comparison across studies. Earlier work used NAFLD or MAFLD definitions, whereas newer studies use MASLD criteria. These populations overlap substantially but are not identical. In addition, hepatic steatosis and fibrosis have been assessed using ultrasound, controlled attenuation parameter, transient elastography, magnetic resonance imaging–proton density fat fraction, laboratory-based scores, or histology. Differences in participant selection and reference standards can materially affect reported associations, AUC values, and optimal cutoffs.

An additional concern is component overlap between the tested marker and the outcome definition. TyG includes fasting triglycerides and glucose, both of which are closely related to the cardiometabolic criteria used to classify MASLD. TyG-BMI, TyG-WC, and TyG-WHtR incorporate anthropometric variables that may also contribute to disease classification. Consequently, part of the observed diagnostic performance may reflect shared components rather than independent identification of liver disease. Future studies should assess TyG-related indices against liver-specific reference standards and report their incremental value beyond established clinical, laboratory, and imaging assessments.

No universal cutoff can currently be recommended. Reported thresholds vary according to age, sex, ethnicity, diabetes status, adiposity, medication use, fasting conditions, and the method used to define steatosis or fibrosis. This variability is especially relevant in lean MASLD and older adults, in whom BMI may not accurately represent adiposity. Direct head-to-head comparisons of TyG, TyG-BMI, TyG-WC, TyG-WHtR, HOMA-IR, and liver-directed non-invasive tests remain limited, preventing a reliable population-specific selection strategy (16, 51).

Finally, evidence for clinically meaningful liver-related prognosis remains insufficient. Several cohorts link TyG-related indices to cardiovascular or renal outcomes, but these findings should not be conflated with prediction of advanced fibrosis, cirrhosis, hepatocellular carcinoma, or liver-related death. Existing liver-outcome studies are promising but require replication in diverse prospective cohorts with standardized outcome definitions and repeated metabolic measurements (52). Until such data are available, TyG-related indices are best regarded as metabolic risk markers that may support, but not replace, liver-specific assessment.

## 5 Future directions for TyG-based risk stratification

The next stage of research should move beyond repeated cross-sectional association studies. Multicentre prospective cohorts are needed across Asian, European, North American, and other populations, with prespecified MASLD definitions and liver-specific reference standards. Fasting status, laboratory assays, anthropometric protocols, medication use, alcohol exposure, and timing of liver assessment should be recorded consistently. Such standardization is necessary before reported TyG thresholds can be compared across studies or considered for clinical use.

Direct head-to-head comparisons should determine which TyG-related index is most informative for a defined clinical purpose. These studies should compare TyG, TyG-BMI, TyG-WC, and TyG-WHtR with HOMA-IR, liver enzymes, established steatosis and fibrosis scores, ultrasound, vibration-controlled transient elastography, and MRI-based measures. Analyses should assess calibration, discrimination, reclassification, and clinical decision benefit, rather than reporting AUC alone. Particular attention is needed for lean MASLD, older adults, people with diabetes, and sex-specific subgroups, in whom body composition and metabolic risk may differ substantially.

Longitudinal studies should also clarify how TyG changes over time. Rather than relying on a single baseline measurement, future cohorts should obtain repeated fasting triglyceride and glucose values at prespecified intervals and use transparent methods to model cumulative exposure and trajectories. The optimal number and timing of measurements remain uncertain. Studies should therefore report these features explicitly and test whether dynamic TyG measures improve prediction beyond baseline TyG and conventional clinical risk factors.

**Figure 3:**
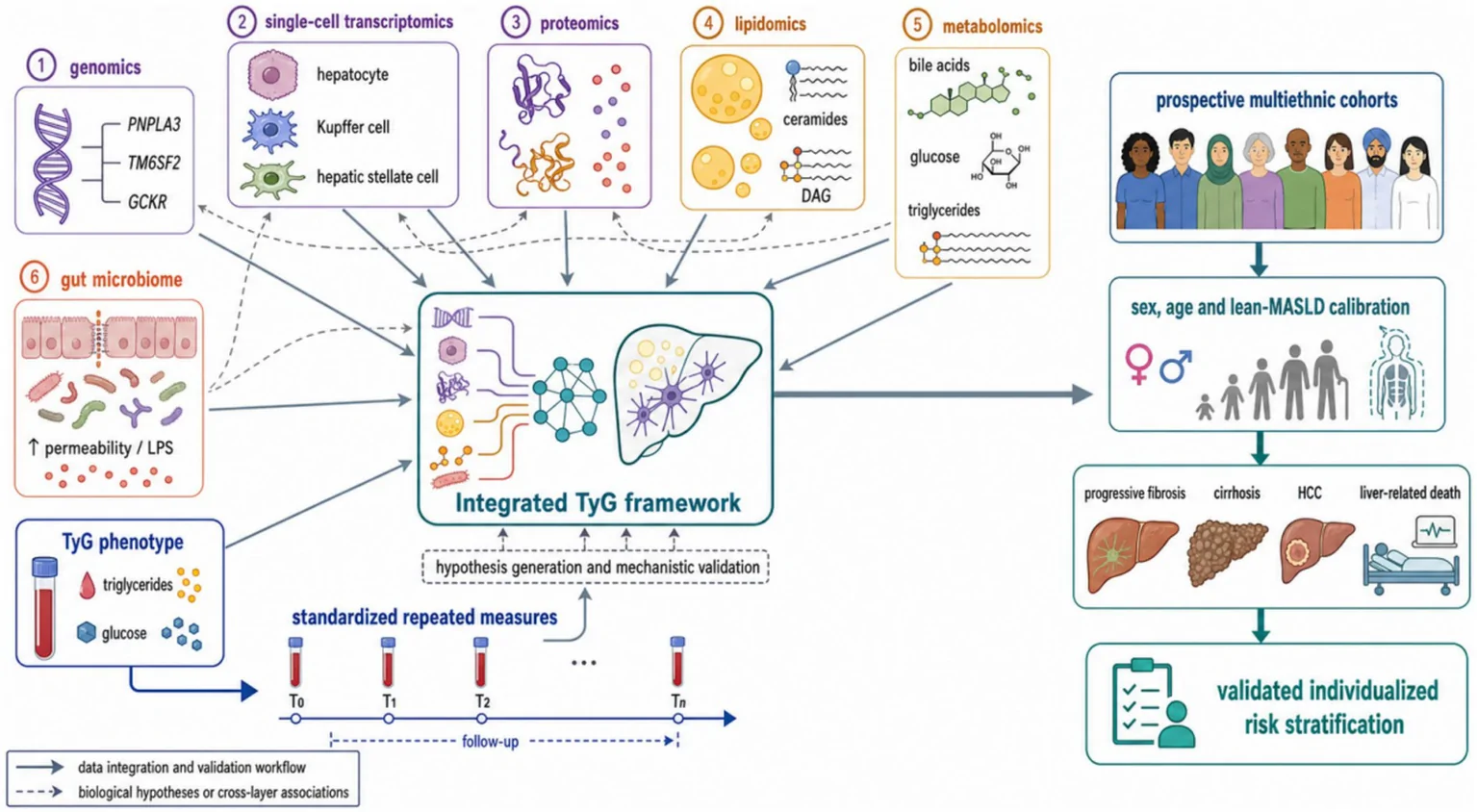
Prospective multi-omics framework for validation of TyG-related risk stratification in MASLD. Genomic susceptibility, single-cell and spatial transcriptomics, proteomics, lipidomics, metabolomics, and gut-microbiome features are integrated with repeated TyG-related measurements and standardized anthropometry. Prospective cohorts should evaluate population-specific calibration and liver-related hard outcomes, including progressive fibrosis, cirrhosis, hepatocellular carcinoma, transplantation, and liver-related death. The framework is intended to generate and validate clinically interpretable risk models rather than imply that current TyG-based tools are already validated for individualized care.

The most important unanswered question is whether TyG-related indices predict liver-specific hard outcomes. Progressive fibrosis, cirrhosis, hepatocellular carcinoma, liver transplantation, and liver-related death should be evaluated separately from cardiovascular and renal outcomes. Multi-omics, genetic, and spatial-transcriptomic approaches may help identify biological subgroups in which TyG-related markers have different meanings or predictive value. However, these approaches should complement, rather than replace, rigorous prospective clinical validation. A future framework should integrate routine metabolic measurements with body-fat distribution, liver-directed tests, genetic susceptibility, and longitudinal outcomes to support precision risk stratification (Figure 3).

## 6 Conclusion

The TyG index is an accessible marker of metabolic dysfunction that may help identify individuals with an unfavorable metabolic profile who warrant further evaluation for MASLD. Its association with hepatic steatosis, fibrosis markers, and extrahepatic outcomes is biologically plausible because fasting triglycerides and glucose reflect several upstream processes linked to insulin resistance and lipid overload. TyG-derived indices may add information when body-fat distribution or inflammation is clinically relevant, but their performance varies across populations and clinical settings. Current evidence supports the use of TyG-related indices as complementary tools for metabolic risk identification and preliminary stratification. They should not be used alone to diagnose MASLD, stage liver disease, distinguish MASH from uncomplicated steatosis, or predict liver-specific hard outcomes. Their clinical interpretation requires attention to disease nomenclature, reference standards, cardiometabolic phenotype, and potential overlap between index components and MASLD diagnostic criteria.

The transition of TyG-related measures from research markers to clinically useful tools will require standardized prospective studies, direct comparison with established metabolic and liver-directed tests, and validation against progressive fibrosis, cirrhosis, hepatocellular carcinoma, and liver-related death. Until then, their most defensible role is to complement, rather than replace, liver-specific assessment.

## Glossary

ABSI: a body shape index
AUC: area under the curve
BMI: body mass index
CAP: controlled attenuation parameter
CI: confidence interval
CRP: C-reactive protein
CTI: C-reactive protein–triglyceride–glucose index
CVD: cardiovascular disease
DAMPs: damage-associated molecular patterns
FFA: free fatty acids
HDL-C: high-density lipoprotein cholesterol
HCC: hepatocellular carcinoma
HOMA-IR: homeostatic model assessment of insulin resistance
HR: hazard ratio
HSCs: hepatic stellate cells
HSL: hormone-sensitive lipase
hs-CRP: high-sensitivity C-reactive protein
IL-6: interleukin-6
IR: insulin resistance
LAP: lipid accumulation product
LPL: lipoprotein lipase
MAFLD: metabolic dysfunction-associated fatty liver disease
MASLD: metabolic dysfunction–associated steatotic liver disease
MASH: metabolic dysfunction–associated steatohepatitis
MetALD: metabolic dysfunction and alcohol-associated liver disease
MRI: magnetic resonance imaging
MRI-PDFF: magnetic resonance imaging–proton density fat fraction
NAFLD: non-alcoholic fatty liver disease
NF-κB: nuclear factor kappa B
NHANES: national health and nutrition examination survey
TLR4: toll-like receptor 4
TGF-β: transforming growth factor beta
ROS: reactive oxygen species
PDGF: platelet-derived growth factor
